# Dynamics of Expression of Programmed Cell Death Protein-1 (PD-1) on T Cells After Allogeneic Hematopoietic Stem Cell Transplantation

**DOI:** 10.3389/fimmu.2019.01034

**Published:** 2019-05-16

**Authors:** Federico Simonetta, Amandine Pradier, Carine Bosshard, Stavroula Masouridi-Levrat, Carole Dantin, Aikaterini Koutsi, Yordanka Tirefort, Eddy Roosnek, Yves Chalandon

**Affiliations:** Division of Hematology, Department of Oncology, Geneva University Hospitals and Faculty of Medicine, University of Geneva, Geneva, Switzerland

**Keywords:** PD-1, checkpoint inhibitors, HSCT, transplantation, exhaustion

## Abstract

Immune exhaustion contributes to treatment failure after allogeneic hematopoietic stem cell transplantation (HSCT) for hematological malignancies. Immune checkpoint blockade, including programmed cell death protein-1 (PD-1) blockade, is a promising strategy to improve the antitumor effect of allogeneic HSCT with high rates of response reported in patients treated for disease relapse. However, severe and sometimes fatal Graft- vs.-Host-Disease (GvHD) has been reported as a complication. Little is known about the dynamics of PD-1 expression on immune effector cells after allogeneic HSCT. In the present study, we analyzed PD-1 expression on T cell subpopulations isolated from 105 allogeneic HSCT recipients. Our analysis revealed a significant increase in proportions of PD-1-expressing CD4 and CD8 T cells early after allogeneic HSCT followed by a progressive normalization of PD-1 expression at CD8 but not CD4 T cell surface. Analysis of co-expression of two other exhaustion markers, 2B4 and CD160, revealed a preferential expansion of PD-1-single positive cells. Moreover, the analysis of granzyme B and perforin expression in PD-1+ and PD-1- CD8 T cells from HSCT recipients did not reveal any impairment in cytotoxic molecules production by PD-1-expressing CD8 T cells. Analyzing the association between clinical factors and the expression of PD-1 on T cells, we identified the use of *in vivo* and/or *ex vivo* T-cell depletion as the factor most strongly associated with elevated PD-1 levels on T cells. Our results extend our knowledge of the regulation of PD-1 expression at T cell surface after allogeneic HSCT, a crucial information for the optimization of post-transplantation PD-1 blocking therapies.

## Introduction

Allogeneic hematopoietic stem cell transplantation (HSCT) is a well-established therapeutic modality for a broad variety of hematological malignancies, unfortunately still associated with a significant risk of cancer relapse. Mechanisms of disease relapse after allogeneic HSCT include resistance to chemotherapy but also escape of tumor cells from the control of the alloreactive immune responses (1). Immune exhaustion of donor-derived immune cells contributes to treatment failure and immune checkpoint blockade is a promising strategy to improve the antitumor effect of the transplantation procedure (2, 3). Several studies reported the association of programmed cell death protein-1 (PD-1) up-regulation at T-cell surface with disease relapse (4–7) and high rates of antitumor responses have been reported in patients treated with programmed cell death protein-1 (PD-1)/programmed death-ligand 1 (PD-L1) blockade administered for disease relapse after allogeneic HSCT (8–10). However, the PD-1/PD-L1 axis plays an important role in maintenance of immune tolerance after allogeneic HSCT as revealed by preclinical studies (11–14) and supported by the high incidence of severe, and in some cases fatal, Graft-vs.-Host-Disease (GvHD) in patients receiving PD-1 blockade for post-transplant relapse (8–10). Little is known about the dynamics of expression of PD-1 at immune effectors' cell surface during immune-reconstitution after transplantation, a crucial information for the optimization of antibody-based PD-1/PD-L1 blockade therapies in the context of allogeneic HSCT. In the present study, we measured PD-1 expression on T cells subpopulations isolated from 105 patients analyzed at different time points after allogeneic HSCT. Our analysis revealed an early and long lasting increase in proportions of PD-1+ CD4 T cells after allogeneic HSCT while we observed only a transient increase in PD-1+ CD8 T cells. Moreover, we identified the use of *in vivo* and/or *ex vivo* T-cell depletion as the clinical factor most strongly associated with elevated PD-1 levels on T cells.

## Patients and Methods

### Study Design and Patients' Characteristics

We prospectively analyzed 148 samples isolated from 105 patients who underwent allogeneic HSCT at our center and were seen at follow-up visits in our outpatient clinic between November 2015 and November 2016. Samples from 24 age-matched, sex-matched healthy blood donors were used as controls. Written informed consent was provided by all individuals enrolled in the study and the study was approved by the ethics committee of the Geneva University Hospitals. Clinical data were retrospectively extracted from patient's medical records. Patients' characteristics are summarized in Table 1. Forty-nine patients (47%) received grafts from an HLA-identical sibling (SIB) and 39 patients (37%) from an HLA-matched unrelated donor (MUD), whereas 3 (3%) patients received grafts from an HLA-mismatched unrelated donor (MMUD). Fourteen patients (13%) received grafts from haploidentical donors. Myeloablative conditioning (MAC) mostly consisted of cyclophosphamide (120 mg/kg) in combination with busulfan (12.8 mg/kg) or total body irradiation (10–12 Gy). Reduced-intensity conditioning (RIC) mainly consisted of fludarabine (120 mg/m^2^) associated with low dose busulfan (6.4 mg/kg) or melphalan (140 mg/m^2^). Most patients (82 patients, 78%) received some form of *in vivo* and/or *ex vivo* T-cell depletion (TCD). *In vivo* TCD by anti-thymocyte globulin (ATG) (Thymoglobulin® 7.5 mg/kg or ATG-Fresenius® 25 mg/kg) was part of conditioning for all patients treated with RIC and for patients receiving grafts from an unrelated donor after a MAC. *Ex vivo* partial TCD was obtained through grafts incubation with alemtuzumab (Campath [Genzyme Corporation, Cambridge, MA]) *in vitro* washed before infusion, administered at day 0, followed on day +1 by an add-back of donor T cells (usually 100 × 10^6^/kg donor T cells) (15). Twenty-nine patients (28%) received ATG alone, 14 patients (13%) *ex vivo* TCD alone and 25 patients (24%) both ATG and *ex vivo* TCD. Fourteen patients (13%) receiving grafts from haploidentical donors were treated with post-transplantation cyclophosphamide as *in vivo* TCD as previously described (16). Graft- vs.-host disease (GvHD) prophylaxis mainly consisted of cyclosporine (for 3 months duration in the absence of GvHD in the case of partial T cell depletion and for 6 months for T cell–replete graft recipients) in combination with either methotrexate, in case of MAC regimen, or mycophenolate mofetil for patients transplanted after RIC. *Ex vivo* TCD graft recipients also received methylprednisolone on days −2 and −1. Donor lymphocyte infusions (DLI) at incremental doses (starting with 5 × 10^5^ CD3/kg for unrelated- and 1 × 10^6^/kg for related donors) were given at 3 months to all patients who had received *ex vivo* TCD grafts with RIC in absence of GvHD. Acute or chronic GvHD was treated with corticosteroids alone or in combination with mycophenolate mofetil and/or cyclosporine.

**Table 1:** Clinical characteristics of HSCT recipients.

		***Patients n=105***	
**AGE**		**YEARS (RANGE**)	
	Age at transplant	50 (18–70)	
**SEX**		**n**	**%**
	Male	65	62
	Female	40	38
**PRIMARY DISEASE**		**n**	**%**
	AML	36	34
	ALL	11	10
	Lymphoma/CLL	16	15
	MDS	15	14
	MPS/MDPS	11	10
	Multiple Myeloma	4	4
	CML	7	7
	Aplastic Anemia	4	4
	Other	1	1
**CELL SOURCE**		**n**	**%**
	PBSC	88	84
	BM	17	16
**DONOR TYPE**		**n**	**%**
	SIB	49	47
	MUD	39	37
	MMUD	3	3
	Haploidentical	14	13
**CONDITIONING REGIMEN**		**n**	**%**
	MAC	58	55
	RIC	47	45
**T-CELL DEPLETION (TCD)**		**n**	**%**
	No TCD	23	22
	ATG	29	28
	Ex vivo	14	13
	ATG + Ex vivo	25	24
	Post-Tx Cy	14	13
**IMMUNOSUPPRESSION**		**n**	**%**
	CSA	4	4
	CSA, MMF	41	39
	CSA, MTX	38	36
	Tacrolimus, MTX	8	8
	Tacrolimus, MMF, Cy	14	13
**CMV SEROSTATUS**		**n**	**%**
	Recipient+	64	61
	Donor+	60	57

*ALL, acute lymphoblastic leukemia; AML, acute myeloid leukemia; ATG, Anti-thymocyte globulin; BM, bone marrow; CLL, chronic lymphocytic leukemia; CML, chronic myeloid leukemia; CSA, cyclosporine; MAC, myeloablative conditioning; MDS, myelodysplastic syndrome; MMF, mycophenolate mofetil; MMUD, mis-matched unrelated donor; MPS, myeloproliferative syndrome; MTX, methotrexate; MUD, matched unrelated donor; PBSC, peripheral blood stem cells; RIC, reduced-intensity conditioning; Post-Tx Cy, post-transplant Cyclophosphamide; SIB, matched related sibling; TCD, T-cell depletion*.

### Flow Cytometry Analysis

Peripheral blood mononuclear cells (PBMCs) were isolated from fresh anticoagulated blood by Ficoll density gradient centrifugation. PBMCs were then stained and analyzed using a ten-color flow cytometry panel. The following conjugated antibodies were used for surface staining: anti-CCR7 (FITC), anti-CD57 (PE-CF594), anti-CD45RA (allophycocyanin-H7), and anti-CD3 (V500) were from BD Biosciences (San Jose, CA); anti-PD-1 (PE), anti-2B4 (PerCp-Cy5.5), anti-CD160 (APC), anti-CD27 (Alexa Fluor 700), anti-CD4 (PECy7), anti-CD8 (BrillantViolet421) were from Biolegend. Intracellular staining for cytotoxic molecules was performed using anti–granzyme B (Alexa Fluor 700, clone GB11 [BD Biosciences]) and anti-Perforin (FITC, clone B-D48 [Diaclone]) on fixed and permeabilized cells following manufacturer's instructions (eBioscience). Samples were acquired on Gallios 3 cell analyzer (BD Biosciences), and data files were analyzed using FlowJo software (Tree Star).

### Statistical Analysis

Statistical analysis was performed using Prism version 7 (GraphPad Inc), R version 3.5.1 (Comprehensive R Archive Network (CRAN) project (http://cran.us.r-project.org) with R studio Version 1.1.453 and EZR version 1.37 (Saitama Medical Center, Jichi Medical University, Saitama, Japan) (17). *P*-values <0.05 were considered statistically significant.

## Results

### PD-1 Expression Is Differentially Regulated at CD4 and CD8 T Cell Surface After Allogeneic HSCT

**Figure 1:**
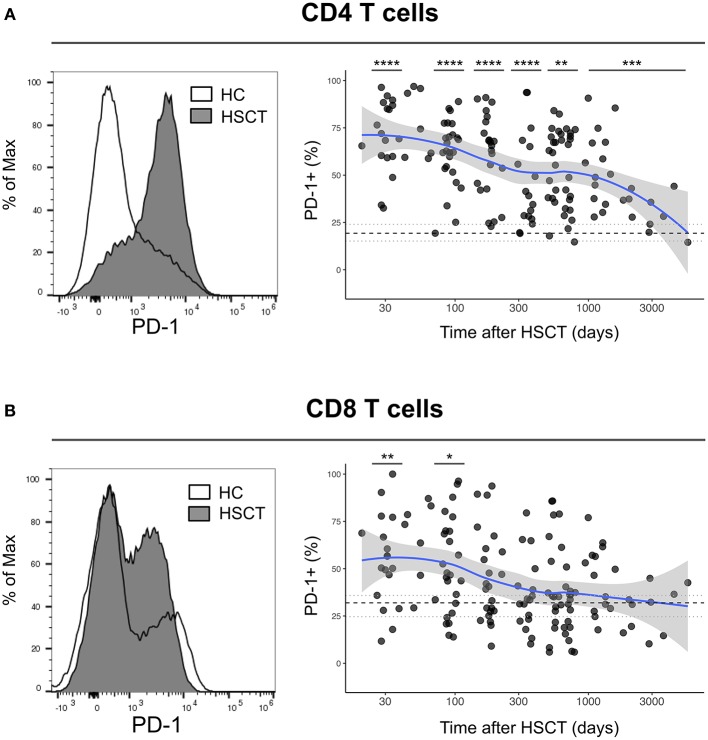
Dynamics of PD-1 expression on CD4 and CD8 T cells after allogeneic HSCT. **(A,B)** Histograms representing PD-1 expression on CD4 **(A)** or CD8 **(B)** T cells from HC (open line) or a HSCT recipient at day 30 post-HSCT (gray filled histogram). Graphs represent proportions of PD-1 expressing CD4 **(A)** or CD8 **(B)** T cells from HSCT recipients and their relationship with time since HSCT. Dark gray dots represent each sample, blue lines represent medians and the gray area represents the confidence interval. Median (black dashed line), 25th and 75th percentiles (gray dotted lines) of PD-1 expressing T cells from HC are represented. The *p-*values of expression in HSCT recipient groups at 1, 3, 6, 12, and >24 months compared with HC are indicated (Mann-Whitney *U*-test; *p < 0.05, **p < 0.01, ***p < 0.001, ****p < 0.0001).

**Figure 2:**
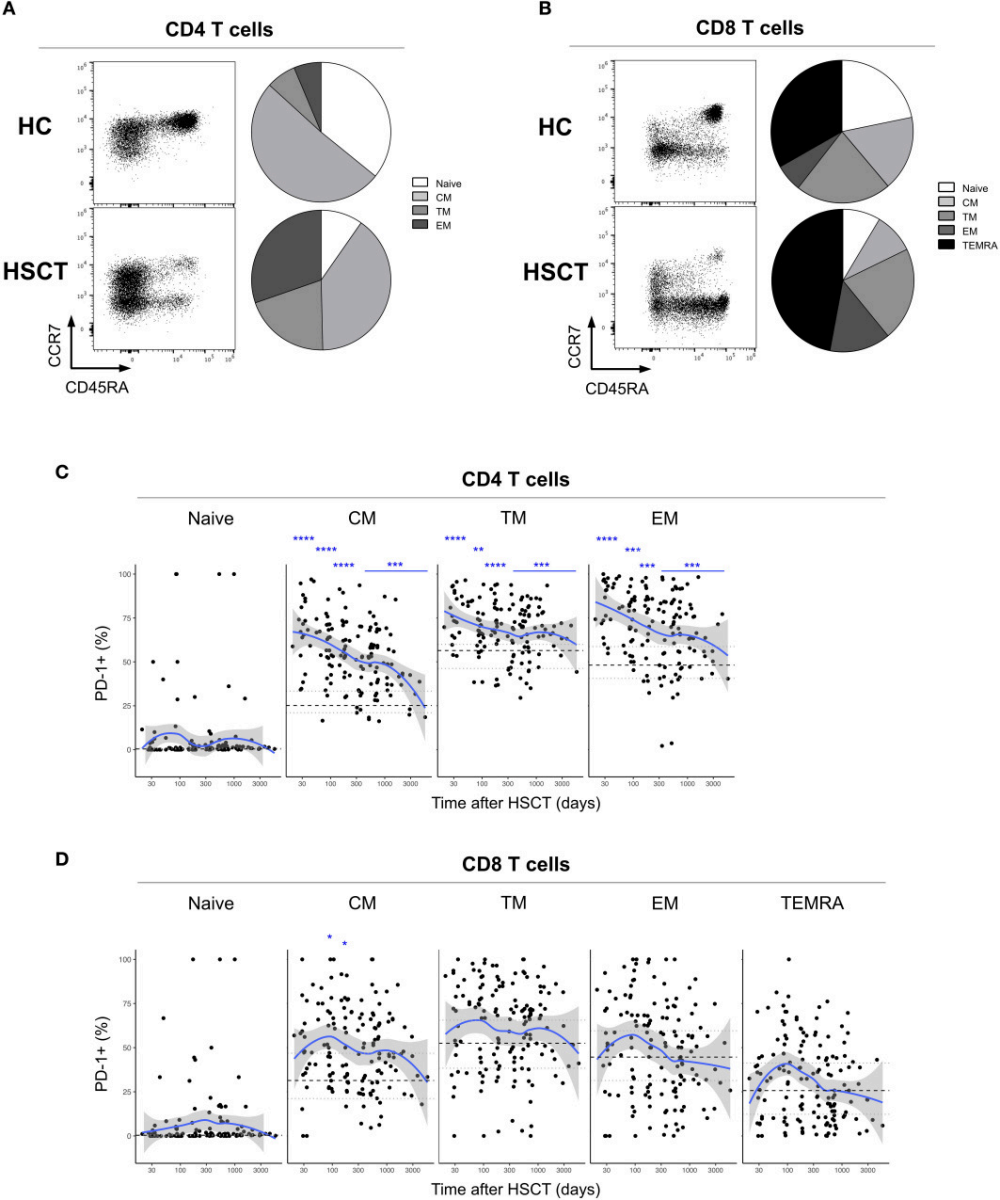
PD-1 expression on CD4 and CD8 T cell subsets after allogeneic HSCT. **(A,B)** Representative FACS dot plots and pie charts representing the proportions of T cell subsets identified based on CD45RA, CCR7, and CD27 expression (Naïve: CD45RA+ CCR7+ CD27+; central memory (CM): CD45RA- CCR7+ CD27+; transitional memory (TM): CD45RA- CCR7- CD27+; effector memory (EM) CD45RA- CCR7- CD27-; effector memory re-expressing CD45RA (TEMRA): CD45RA+ CCR7- CD27-). Different colors correspond to subsets as indicated. **(C,D)** proportions of PD-1 expressing CD4 **(C)** or CD8 **(D)** T cell subsets from HSCT recipients (black filled circles) and their relationship with time since HSCT. Blue lines represent medians and the gray area represents the confidence interval. Median (black dashed line), 25th and 75th percentiles (gray dotted lines) of PD-1 expressing T cells from HC are represented. The *p-*values of expression in HSCT recipient groups at 1, 3, 6, 12, and >12 months compared with HC are indicated (Mann-Whitney *U*-test; *p < 0.05, **p < 0.01, ***p < 0.001, ****p < 0.0001).

We first analyzed the expression of PD-1 at the surface of CD4 and CD8 T cells from healthy control subjects (HC) and from patients after HSCT. HSCT recipients displayed significantly higher proportions of PD-1 expressing CD4 [median (range), 61% (15–97%)] and CD8 [38% (6–100%)] T cells compared with HC [CD4 19% (7–31%), *p* < 0.0001; CD8 32% (8–56%), *p* = 0.0124] (Figures 1A,B). Given the severe immune homeostasis alteration present immediately after HSCT because of the severe lymphopenia and the pro-inflammatory environment secondary to the conditioning regimens, we next investigated whether the observed increase in PD-1 expression at T cell surface was only a transient or rather a sustained, long-lasting T cell abnormality after HSCT. We found a significant negative correlation between the time elapsed since transplantation and the proportion of PD-1 expressing CD4 (*r* = −0.3755, *p* < 0.0001; Figure 1A) and CD8 (*r* = −0.3176, *p* < 0.0001; Figure 1B) T cells. Interestingly, we observed a significantly higher proportion of PD-1+ CD4 T cells isolated from HSCT recipients compared with HC at all-time points studied including patients studied more than 5 years post-transplantation (Figure 1A). Conversely, CD8 T cells isolated from patients at 1 and 3 months post-transplantation exhibited increased levels of PD-1 expression compared to healthy controls while we failed to detect any significant difference between HSCT and HC at later time points (Figure 1B). Immune reconstitution after HSCT is associated with an altered distribution of T cell subsets, with an over-representation of effector/memory cells and a reduction in naïve cells (Figures 2A,B). As PD-1 is constitutively expressed at higher proportions on effector/memory cells compared to naïve cells, we analyzed PD-1 expression in T cell subpopulations to determine whether the observed increase in PD-1 positive T cells in HSCT recipients is a mere consequence of the increased proportions of effector/memory subsets or an actual up-regulation of PD-1 at T cell subsets surface. When T cell subsets heterogeneity was taken into account, we observed higher proportions of PD-1 expressing cells in all effector/memory CD4 T cell subsets from HSCT recipients, including CD45RA- CCR7+ CD27+ central memory (CM), CD45RA- CCR7- CD27+ transitional memory (TM) and CD45RA- CCR7- CD27- effector memory (EM) (Figure 2C). Interestingly, such increase was sustained during time, PD-1 expression being significantly higher in HSCT recipients even after more than 24 months since transplantation. Conversely, among CD8 T cell subsets we only observed a significant PD-1 up-regulation within CD8 CM T cells from HSCT recipients during the first 6 months after transplantation, while no difference was observed in TM, EM and effector memory re-expressing CD45RA (TEMRA) at any of the time points studied (Figure 2D). Collectively these results point to different dynamics of PD-1 expression in CD4 and CD8 T cells after HSCT, CD4 T cells up-regulating PD-1 expression on all effector/memory cells for more than 2 years after transplantation, CD8 T cells only transiently expressing higher proportions of PD-1 as a consequence of the effector/memory skewing after transplantation as well as of a transient PD-1 overexpression on CM cells.

### PD-1 Expression at T Cell Surface After HSCT Is Only Partially Associated With Co-Expression of Other Exhaustion Markers

**Figure 3:**
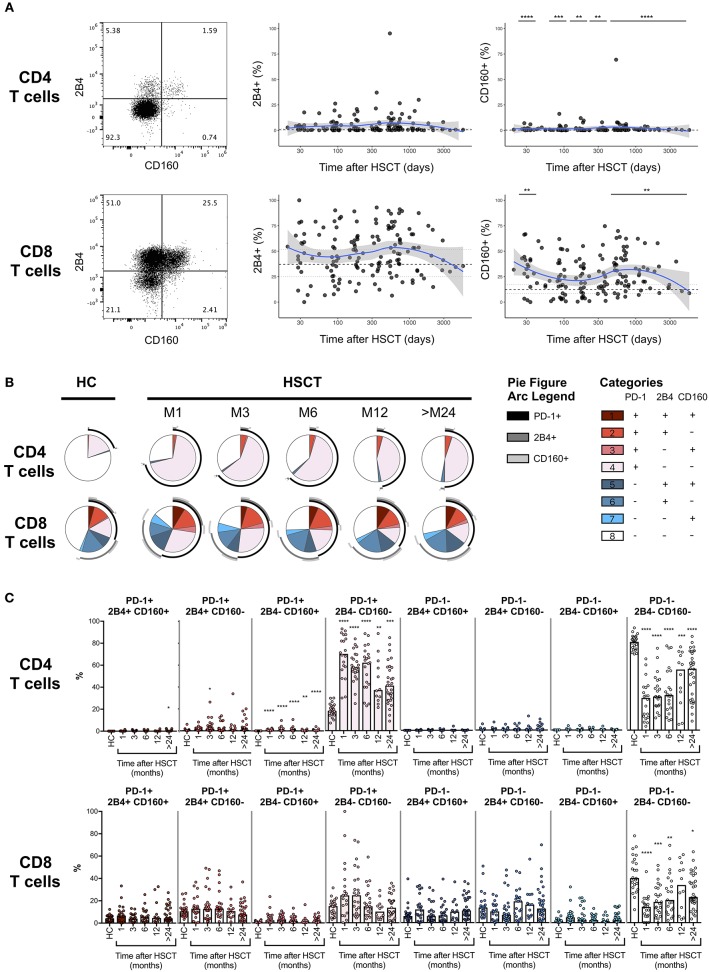
Co-expression of PD-1, 2B4, and CD160 on CD4 and CD8 T cells after allogeneic HSCT. **(A)** Representative dot plots (left panels) and proportions of 2B4 and CD160 expressing CD4 or CD8 T cell subsets from HSCT recipients (black filled circles) and their relationship with time since HSCT. Blue lines represent medians and the gray area represents the confidence interval. Median (black dashed line), 25th and 75th percentiles (gray dotted lines) of 2B4 and CD160 expressing T cells from HC are represented. The *p*-values of expression in HSCT recipient groups at 1, 3, 6, 12, and >24 months compared with HC are indicated (Mann-Whitney *U*-test; *p < 0.05, **p < 0.01, ***p < 0.001, ****p < 0.0001). **(B)** Pie charts representing co-expression of 2B4 and CD160 by PD-1 negative (blue gradient colors) and PD-1 positive (red gradient color) CD4 (upper pies) or CD8 (lower pies) T cells. Arcs represent single molecules expression accordingly to the indicated colors. **(C)** Proportions of PD-1, 2B4, and CD160 co-expressing CD4 or CD8 T cell subsets in HC and HSCT recipients. The *p*-values of expression in HSCT recipient groups at indicated time compared with HC are shown (Kruskal-Wallis test; *p < 0.05, **p < 0.01, ***p < 0.001, ****p < 0.0001).

PD-1 represents the most accepted marker of T cell exhaustion identified so far and the only one being a direct target of approved immunotherapeutic strategies. However, PD-1 is also up-regulated during T cell activation and co-expression with other surface markers, including 2B4 and CD160, seems to better identify truly exhausted cells (18–20). We, therefore, assessed the expression of 2B4 and CD160 on T cells after HSCT (Figure 3A, left panels). No difference in the expression of 2B4 was detected on CD4 or CD8 T cells from HSCT recipients compared with HC (Figure 3A, middle panels). Conversely, we observed slight but significant up-regulation of CD160 on T cells from HSCT recipients compared to HC, on CD4 at all-time point studied and on CD8 T cells recovered at 1 month as well as more than 24 months post-transplantation (Figure 3A, right panels). We next analyzed the co-expression of the exhaustion markers PD-1, 2B4, and CD160 in T cells from HSCT recipients. As shown in Figures 3B,C, we observed a strong increase in the proportion of single positive PD-1 CD4 T cells with only limited 2B4 and CD160 co-expression (Figures 3B,C, upper panels). Interestingly, the expansion of this PD-1-single positive CD4 T cells was greatest at early time points but still persisted more than 24 months after transplantation. Conversely, the increased PD-1 expression in CD8 T cells was secondary to the expansion of both PD-1 single positive cells and PD-1 cells co-expressing 2B4 and/or CD160 (Figures 3B,C, lower panels). Importantly, this analysis confirmed the transient nature of the increase in PD-1 expressing CD8 T cells after transplantation (Figures 3B,C). Collectively, these results confirm the minimal and transient increase in PD-1 expression on CD8 T cell subsets and show a sustained expansion of single positive PD-1-expressing CD4 T cells without any accompanying up-regulation of the other T cell exhaustion markers 2B4 and CD160.

### PD-1 Expression at CD8 T Cell Surface Is Not Associated With Impaired Cytotoxic Molecules Production After Allogeneic HSCT

To assess whether PD-1 expression at CD8 T cell surface was potentially associated with functional impairment, we analyzed the expression of the cytotoxic molecules granzyme B and perforin in CD8 T cells isolated from an independent cohort of 10 healthy controls and 10 patients within 12 months since HSCT. As shown in Supplemental Figure 1A, no difference was observed between PD-1+ and PD-1- CD8 T cells in terms of perforin and granzyme B expression. Interestingly, both PD-1+ and PD-1- CD8 T cells from HSCT recipients expressed higher levels of perforin and granzyme B compared with their counterparts from HC (Supplemental Figure 1A) potentially reflecting the different distribution in CD8 T cell subsets in HSCT recipients. We therefore assessed perforin and granzyme B expression within CD8 effector and memory T cell subsets, namely CM, EM and TEMRA. We did not perform this analysis within the naïve CD8 T cell compartment as, in agreement with data reported in Figure 2D, this cell subset was virtually deprived of PD-1+ cells. As represented in Supplemental Figures 1B–D, we did not observe any reduction in perforin or granzyme B content in PD-1+ CM, EM and TEMRA CD8 T cells compared to their PD-1- counterparts. Interestingly, PD-1+ CM CD8 T cells displayed significantly higher levels of granzyme B compared to PD-1- CM CD8 T cells both in healthy controls and HSCT recipients (Supplemental Figure 1B). Collectively, these data do not support the existence of any impairment in cytotoxic molecules production in PD-1+ CD8 T cells recovered from both healthy controls and HSCT recipients.

### T-Cell Depletion Induces Further PD-1 Up-Regulation on T Cells After Allogeneic HSCT

**Figure 4:**
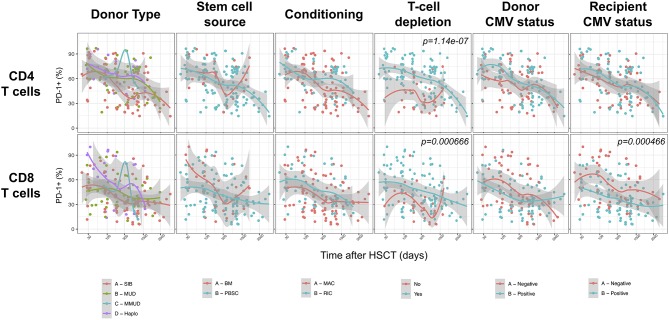
Impact of clinical factors on PD-1 expression at CD4 and CD8 T cells surface after allogeneic HSCT. Comparison of PD-1 expression on CD4 and CD8 T cells from HSCT recipients depending on donor type, stem cell source, conditioning, T-cell depletion and donor/recipient CMV status. The *p*-values resulting from the multivariable linear regression analysis are indicated when significant.

**Figure 5:**
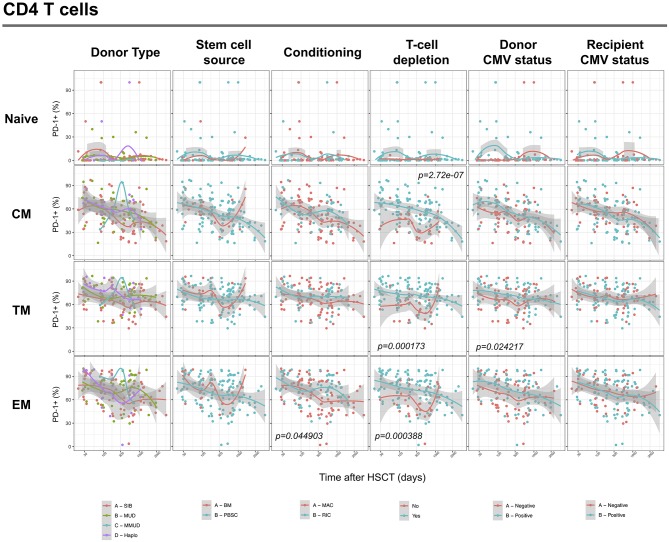
Impact of clinical factors on PD-1 expression at CD4 T cell subsets surface after allogeneic HSCT. Comparison of PD-1 expression on CD4 T cell subsets from HSCT recipients depending on donor type, stem cell source, conditioning, T-cell depletion and donor/recipient CMV status. The *p*-values resulting from the multivariable linear regression analysis are indicated when significant.

**Figure 6:**
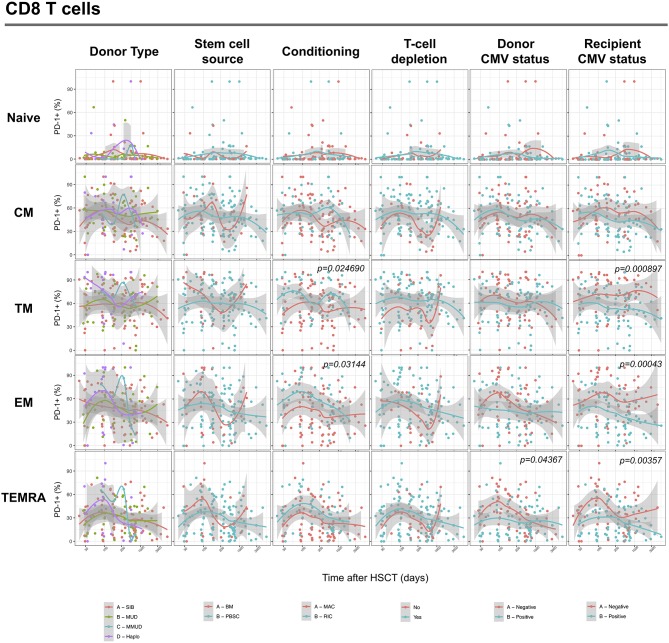
Impact of clinical factors on PD-1 expression at CD8 T cell subsets surface after allogeneic HSCT. Comparison of PD-1 expression on CD8 T cell subsets from HSCT recipients depending on donor type, stem cell source, conditioning, T-cell depletion and donor/recipient CMV status. The *p-*values resulting from the multivariable linear regression analysis are indicated when significant.

**Figure 7:**
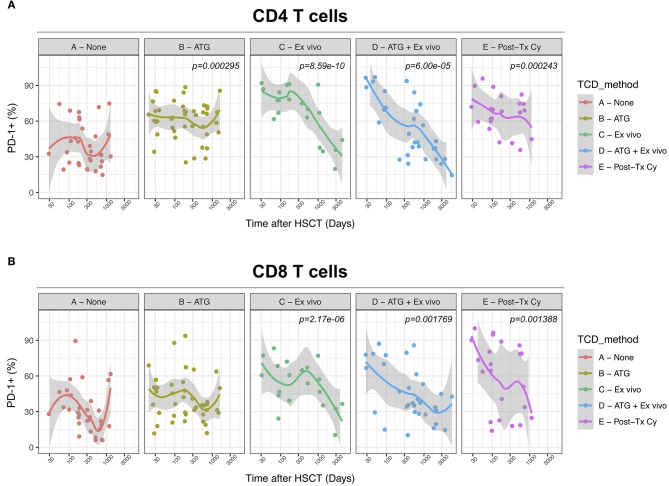
Impact of different methods of T-cell depletion on PD-1 expression on CD4 and CD8 T cells after allogeneic HSCT. Comparison of PD-1 expression on CD4 **(A)** and CD8 **(B)** T cells from HSCT recipients receiving the indicated type of T-cell depletion. The *p*-values resulting from the multivariable linear regression analysis are indicated when significant.

We next assessed the impact of clinical factors on PD-1 expression levels on T cells performing a multivariable linear regression analysis taking into account time elapsed since transplantation, stem cell source, donor type, conditioning regimen, recipient/donor CMV serostatus and use of T-cell depletion. This analysis confirmed the inverse relationship between the time elapsed since HSCT and PD-1 expression on both CD4 (*p* < 0.0001) and CD8 (*p* = 0.007) T cells. We found no association of donor/recipient matching, stem cell source, type of conditioning regimen and donor CMV serostatus with PD-1 expression on CD4 and CD8 T cells (Figure 4). Positive recipient CMV serostatus was associated with a statistically significant reduction of PD-1 expression on CD8 (estimate ± Standard Error, −14 ± 4; *p* = 0.0005) but not CD4 T cells (Figure 4). Interestingly, use of T-cell depletion was strongly associated with increased PD-1 expression on both CD4 (23 ± 4; *p* < 0.00001) and CD8 (16 ± 4.5; *p* = 0.0007) (Figure 4). Given the altered cell subsets distribution after HSCT (Figures 2A,B), we performed similar analysis on CD4 (Figure 5) and CD8 (Figure 6) T cell subsets. Such analysis confirmed the significant impact of T-cell depletion on effector and memory CD4 T cell compartments (Figure 5) while failed to confirm this association in CD8 T cell subsets (Figure 6) suggesting that in this latter case T-cell depletion is affecting T cell subsets distribution more than PD-1 expression itself. Conversely, this analysis confirmed the association between a negative CMV serostatus in the recipient and higher levels of PD-1 in CD8 TM, EM and TEMRA cells (Figure 6). To further investigate the association between T-cell depletion and PD-1 expression at T cell surface, we analyzed the impact of different TCD methods. Use of *in vivo* TCD by ATG administration alone was associated with increased expression of PD-1 at CD4 (Figure 7A) but not CD8 T cell surface (Figure 7B). Conversely, *ex vivo* TCD, alone or in combination with *in vivo* ATG administration, was associated with strong PD-1 up-regulation on both CD4 and CD8 T cells. Finally, use of *in vivo* TCD by administration of post-transplantation cyclophosphamide in the context of HSCT from haploidentical donors was significantly associated with increased PD-1 expression on CD4 and CD8 T cells (Figures 7A,B). We performed a similar analysis to evaluate the potential contribution of immune suppression administered for GvHD prophylaxis on PD-1 expression (Supplemental Figure 2). This analysis revealed a weak, although significant positive association between the use of methotrexate-containing prophylaxis regimens and PD-1 expression in CD4 and CD8 T cells (Supplemental Figure 2). Collectively, these results demonstrate a strong association between the use of TCD and PD-1 upregulation on T cells after HSCT, suggesting a role for post-transplant lymphopenia in the regulation of PD-1 expression at T cell surface.

### Limited Influence of GvHD on PD-1 Surface Expression on T Cells After Allogeneic HSCT

**Figure 8:**
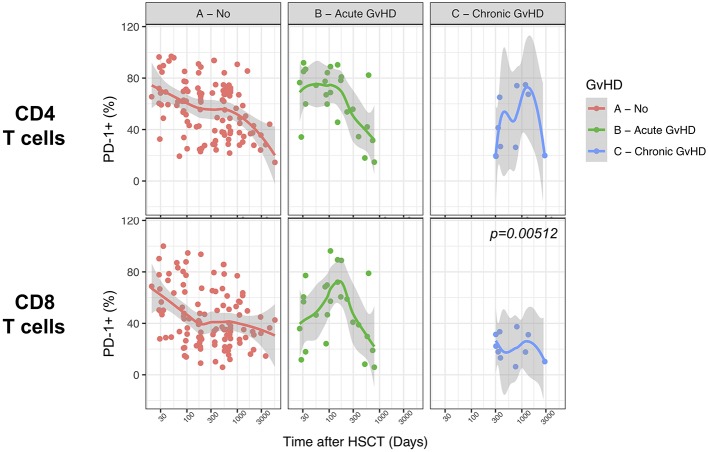
Impact of acute and chronic GvHD on PD-1 expression on CD4 and CD8 T cells after allogeneic HSCT. Comparison of PD-1 expression on CD4 and CD8 T cells from HSCT recipients with no GvHD, acute GvHD or chronic GvHD. The *p*-values resulting from the multivariable linear regression analysis are indicated when significant.

We finally analyzed the relationship between PD-1 expression at T cell surface and post-transplant complications. We failed to detect any significant difference in PD-1 expression on CD4 and CD8 T cells from patients with active acute GvHD (Figure 8). Conversely, chronic GvHD was associated with a slight but significant reduction in PD-1 expression at CD8 but not CD4 T cell surface (Figure 8). The limited number of patients displaying an active disease relapse at the time of sampling in our cohort (*n* = 5) precluded any assessment of the relationship between this complication and PD-1 expression. Collectively, these results point to only a minor impact of GvHD on PD-1 expression levels on T cells after allogeneic HSCT.

## Discussion

Our prospective study revealed a significant increase in the proportions of PD-1-expressing CD4 and CD8 T cells at early phases after allogeneic HSCT followed by a progressive normalization of PD-1 expression at CD8 but not CD4 T cell surface. This expression pattern suggests that the targets of PD-1 blocking treatments might differ depending on time since transplantation at which this treatment is administered. According to our results, PD-1 blockade during the early time after transplantation would affect both CD4 and CD8 T cells. Conversely, administration at later time points, when PD-1 expression at CD8 T cell surface already returns to normal levels, would probably result mainly in anti-PD-1 binding on CD4 T cells. It is therefore tempting to speculate that the effects of PD-1/PDL-1 blockade might differ depending on the time of administration since HSCT with early administration eliciting both helper and cytotoxic T-cell responses while later administration essentially activating the CD4 helper compartment. Similarly, we can speculate that the risk of GvHD exacerbation following PD-1 blockade might differ depending on the time of administration, later administration being potentially associated with higher risk of GvHD development as a result of CD4 rather than CD8 stimulation. Detailed analysis of PD-1 expression at T cell surface during clinical trials investigating safety and efficacy of PD-1 blockade after allogeneic HSCT will address this question.

In our study, we analyzed the relationship between clinical factors at time of HSCT and PD-1 expression. Multivariable linear regression analysis revealed the strongest association between T-cell depletion (TCD) and PD-1 expression at T cell surface, pointing to post-transplant lymphopenia as a major driver of PD-1 upregulation after HSCT. Our results are in agreement with a previous study by Beider et al. reporting higher levels of PD-1 expression on CD4+ CD25+ T cells isolated from HSCT recipients receiving ATG as part of the conditioning regimen compared with patients receiving no TCD (21). Interestingly the observed up-regulation of PD-1 in our cohort was consistent in all methods of TCD including *ex vivo* and *in vivo* TCD, employing either ATG or post-transplant cyclophosphamide. We can hypothesize that PD-1 up-regulation could contribute to the higher risk of relapse reported after TCD. In this case, HSCT recipients receiving TCD grafts could represent a patient group that might particularly benefit from PD-1 blocking strategies. These are hypotheses that need further investigation. Importantly, our multivariable analysis failed to show any association between donor/recipient HLA-matching with PD-1 expression, suggesting that alloreactivity is not necessary for PD-1 upregulation after HSCT. This result, together with the aforementioned impact of lymphopenia on PD-1 expression, is consistent with previous preclinical (22) and clinical (23) studies revealing PD-1 upregulation even after autologous HSCT.

PD-1 is up-regulated at T cell surface during activation (24) and in some cases has been shown to reflect immune activation rather than cell exhaustion in contexts of acute viral infection (25), cancer (26) and inflammatory disorders (27). Whether PD-1 expression at T cell surface after allogeneic HSCT reflects a true exhausted status or rather identifies activated cells remains unclear. The analysis of co-expression of other exhaustion markers, namely CD160 and 2B4, failed to detect any preferential expansion of cells co-expressing two or more exhaustion markers, supporting the hypothesis that the single-positive PD-1 compartment is mainly constituted of activated rather than exhausted T cells. A complementary analysis performed on a subsets of patients failed to show any differences between PD-1+ and PD-1- CD8 T cells in terms of effector cytotoxic molecules expression further supporting the hypothesis that, after allogeneic HSCT, PD-1 expression might mainly reflect a general cellular activation status. Further studies, characterizing PD-1 expressing cells from HSCT recipients at the transcriptomic (27) and epigenomic (28) level, will help to solve this question.

Our study has several limitations. First, the proportion of patients displaying an active disease relapse at time of sampling in our cohort was too small to draw any solid conclusions about the association between PD-1 expression at T-cell surface and disease relapse after HSCT that has been reported by several independent groups (4–6). Second, our analysis is limited to lymphocytes isolated from peripheral blood of HSCT recipients. The dynamics of PD-1 expression reported here might therefore not reflect the pattern of expression at tissue sites. In particular, bone marrow infiltrating T cells might display different PD-1 expression, more specific to this anatomic compartment. Several reports demonstrated in mice (12, 29) and humans (7, 30) that CD8 T cells infiltrating the bone marrow after allogeneic HSCT express significantly higher levels of PD-1 at their surface compared to peripheral blood cells, a difference that might derive from the interaction with the micro-environment and/or with tumor cells in case of disease persistence or relapse. In a very elegant study comparing bone marrow-infiltrating T cells from HSCT recipients displaying relapse of acute myeloid leukemia after transplantation and from patients maintaining complete remission, Noviello et al. (7) recently reported higher proportions of T cells expressing inhibitory receptors, including PD-1, in relapsing patients than in patients maintaining complete remission. Moreover, by studying PD-1 co-expression with co-stimulatory molecules and T-box transcription factors, the authors further show that PD-1+Eomes+T-bet– phenotype in bone marrow-infiltrating CD8 T Memory Stem cells allows prediction of disease relapse (7).

In summary, our results indicate that the dynamics of PD-1 expression on T cells after allogeneic HSCT are differentially regulated in CD4 and CD8 T cells. These results suggest potentially different cellular targets, and consequently effects, depending on the time since transplantation at which PD-1/PD-L1 blockade may be used. Moreover, we identify the use of T-cell depletion as a major contributor to the induction of PD-1 upregulation on T cells after allogeneic HSCT. These results may have important implications for the optimization of PD-1/PD-L1 blocking therapies and stress the importance of performing detailed immune-monitoring studies during future clinical trials evaluating PD-1/PD-L1 blockade after allogeneic HSCT.

## Ethics Statement

This study was carried out in accordance with the recommendations of the Commission Cantonale d'Ethique de la Recherche Scientifique de Genève with written informed consent from all subjects. All subjects gave written informed consent in accordance with the Declaration of Helsinki. The protocol was approved by the Commission Cantonale d'Ethique de la Recherche Scientifique de Genève (Protocol #12–138).

## Author Contributions

FS conceived and coordinated the study, analyzed the data, prepared the figures and wrote the manuscript. AP and CB performed the experiments and critically revised the manuscript. SM-L, CD, AK, and YT collected the clinical data and critically revised the manuscript. ER and YC conceived the study, provided overall guidance, and edited the manuscript.

### Conflict of Interest Statement

The authors declare that the research was conducted in the absence of any commercial or financial relationships that could be construed as a potential conflict of interest.

## Supplementary Material

Supplemental Figure 1 Perforin and Granzyme B expression in PD-1+ and PD-1- CD8 T cells. Proportions of perforin (left panels) and granzyme B (right panels) expressing cells among PD-1+ and PD-1- CD8 T cells **(A)** and CD8 T cell subsets **(B–D)** from HC (white filled dots) or HSCT recipients (black filled dots). The *p*-values are indicated when significant (Wilcoxon signed-rank test).

Supplemental Figure 2 Impact of different immunosuppressive drugs for GvHD prophylaxis on PD-1 expression on CD4 and CD8 T cells after allogeneic HSCT. Comparison of PD-1 expression on CD4 (**Upper Panel**) and CD8 (**Lower Panel**) T cells from HSCT recipients receiving the indicated GvHD prophylaxis. The *p*-values resulting from the multivariable linear regression analysis are indicated when significant.
